# Coocclusion of Helicoverpa armigera Single Nucleopolyhedrovirus (HearSNPV) and Helicoverpa armigera Multiple Nucleopolyhedrovirus (HearMNPV): Pathogenicity and Stability in Homologous and Heterologous Hosts

**DOI:** 10.3390/v14040687

**Published:** 2022-03-26

**Authors:** Maite Arrizubieta, Oihane Simón, Adriana Ricarte-Bermejo, Miguel López-Ferber, Trevor Williams, Primitivo Caballero

**Affiliations:** 1 Institute for Multidisciplinary Research in Applied Biology, Universidad Pública de Navarra, 31006 Pamplona, Spain; maitearrizubieta@hotmail.com (M.A.); oihane.simon@unavarra.es (O.S.); adriana.ricarte@unavarra.es (A.R.-B.); 2 Hydrosciences Montpellier, Université de Montpellier, IMT Mines Ales, IRD, CNRS, 30319 Ales, France; miguel.lopez-ferber@mines-ales.fr; 3 Instituto de Ecología AC (INECOL), Xalapa 91073, Veracruz, Mexico; 4 Departamento de Investigación y Desarrollo, Bioinsectis SL, 31110 Noain, Spain

**Keywords:** old world cotton bollworm, HearNPV, Baculoviridae, *Mamestra brassicae*, *Spodoptera frugiperda*, occlusion derived virion, host range, virus insecticide

## Abstract

Helicoverpa armigera single nucleopolyhedrovirus (HearSNPV) is a virulent pathogen of lepidopterans in the genera *Heliothis* and *Helicoverpa*, whereas Helicoverpa armigera multiple nucleopolyhedrovirus (HearSNPV) is a different virus species with a broader host range. This study aimed to examine the consequences of coocclusion of HearSNPV and HearMNPV on the pathogenicity, stability and host range of mixed-virus occlusion bodies (OBs). HearSNPV OBs were approximately 6-fold more pathogenic than HearMNPV OBs, showed faster killing by approximately 13 h, and were approximately 45% more productive in terms of OB production per larva. For coocclusion, *H. armigera* larvae were first inoculated with HearMNPV OBs and subsequently inoculated with HearSNPV OBs at intervals of 0–72 h after the initial inoculation. When the interval between inoculations was 12–24 h, OBs collected from virus-killed insects were found to comprise 41–57% of HearSNPV genomes, but the prevalence of HearSNPV genomes was greatly reduced (3–4%) at later time points. Quantitative PCR (qPCR) analysis revealed the presence of HearSNPV genomes in a small fraction of multinucleocapsid ODVs representing 0.47–0.88% of the genomes quantified in ODV samples, indicating that both viruses had replicated in coinfected host cells. End-point dilution assays on ODVs from cooccluded mixed-virus OBs confirmed the presence of both viruses in 41.9–55.6% of wells that were predicted to have been infected by a single ODV. A control experiment indicated that this result was unlikely to be due to the adhesion of HearSNPV ODVs to HearMNPV ODVs or accidental contamination during ODV band extraction. Therefore, the disparity between the qPCR and end-point dilution estimates of the prevalence of mixed-virus ODVs likely reflected virus-specific differences in replication efficiency in cell culture and the higher infectivity of pseudotyped ODVs that were produced in coinfected parental cells. Bioassays on *H. armigera*, *Spodoptera frugiperda* and *Mamestra brassicae* larvae revealed that mixed-virus OBs were capable of infecting heterologous hosts, but relative potency values largely reflected the proportion of HearMNPV present in each mixed-virus preparation. The cooccluded mixtures were unstable in serial passage; HearSNPV rapidly dominated during passage in *H. armigera* whereas HearMNPV rapidly dominated during passage in the heterologous hosts. We conclude that mixed-virus coocclusion technology may be useful for producing precise mixtures of viruses with host range properties suitable for the control of complexes of lepidopteran pests in particular crops, although this requires validation by field testing.

## 1. Introduction

The cotton bollworm, *Helicoverpa armigera* (Hübner) (Lepidoptera: Noctuidae), is an invasive insect pest that feeds on many crops in Eurasia, Africa and Oceania and is currently spreading through South America and the Caribbean region [1]. Frequent application of broad-spectrum synthetic insecticides has resulted in high levels of resistance in this pest [2]. Alternative control methods include the use of nucleopolyhedrovirus-based insecticides that are both effective and safe for insect natural enemies, pollinators and other non-target organisms [3,4].

Lepidopteran nucleopolyhedroviruses (NPVs) (genus *Alphabaculovirus*; Family Baculoviridae) are double stranded DNA viruses. The rod-like nucleocapsids are enveloped singly (single nucleocapsid NPVs, SNPV) or in groups (multiple nucleocapsid NPVs, MNPV) to form occlusion derived virions (ODVs) [5]. The ODVs are occluded within a protein matrix consisting mostly of polyhedrin to form occlusion bodies (OBs) that are responsible for insect-to-insect horizontal transmission and persistence of the virus in the environment [6,7]. Infection occurs when larvae consume OB-contaminated foliage. OBs dissolve in the alkaline conditions of the larval midgut releasing ODVs that cross the peritrophic membrane to infect midgut epithelial cells [8]. Following replication or repackaging in midgut cells, nucleocapsids bud through the basal membrane and disperse in the form of budded virions to infect the cells of other tissues [9]. Later in infection OBs are produced in the nucleus. A few days after initial infection larvae die and undergo liquification on the upper parts of plants, releasing millions of OBs for the following transmission cycle [10].

Natural isolates of NPVs are genotypically diverse [11,12]. As multinucleocapsid ODVs can envelope several nucleocapsids, they can transmit different genotypes of the same virus [13,14], or even different virus species [15], during infection of larval midgut cells. Similarly, during the systemic phase of infection, cells can be infected by several budded virions, each carrying a single genome [16], so that diversity is transmitted within individual insects [17,18,19], during the temporal window (~16 h) for which cells are susceptible to coinfection by budded virions [20,21].

NPVs with both the multiple and single nucleocapsid morphotype have been characterized from a small number of host species, including *Helicoverpa armigera* [22,23,24,25,26,27]. In the case of *H. armigera*, isolates of HearSNPV from geographically distinct locations are highly similar at the genome level [28,29,30,31,32,33,34]. Isolates of HearSNPV have a narrow host range [35], which is limited to species in the genera *Helicoverpa* and *Heliothis* [22]. In contrast, isolates of HearMNPV show greater genetic diversity across geographical locations [27,36], and infect a broader range of hosts, including species outside of the *Helicoverpa*/*Heliothis* complex [37,38,39]. In this sense HearMNPV resembles the closely related broad host range NPVs from *Mamestra brassicae* (MbMNPV) and *Mamestra configurata* (MacoNPV-B) [30]. At the genomic level, HearSNPV and HearMNPV are quite distantly related within the group II NPVs [36]. In terms of their insecticidal properties, HearSNPV is more virulent and typically kills larvae faster than HearMNPV. Due to these clear genetic and phenotypical differences HearSNPV and HearMNPV are classified as different baculovirus species [5]. Conventionally, the official abbreviation for HearSNPV is HearNPV, but the “S” designation is used throughout this text for clarity.

When considering developing an NPV-based insecticide against species in the *Helicoverpa*/*Heliothis* complex, an isolate of HearSNPV should be selected due to its high virulence against these pests [3,40,41]. However, where a crop is also attacked by other lepidopteran pests, the use of an NPV with a wider host range is desirable. The use of HearMNPV might address this problem. A previous study by our team demonstrated that different species of viruses could coinfect and replicate simultaneously in cells of a permissive host, resulting in cooccluded mixtures of viruses with unique insecticidal properties [15]. Therefore, the present study aimed to examine the consequences of coinfection by HearSNPV and HearMNPV on the phenotype, stability and host range characteristics of cooccluded mixed-virus preparations.

## 2. Materials and Methods

### 2.1. Insect Colonies, Viruses and Cell Line

Larvae of *Helicoverpa armigera*, *Mamestra brassicae* and *Spodoptera frugiperda* were obtained from laboratory colonies continuously reared at the Universidad Pública de Navarra (UPNA) (25 ± 1 °C, 70 ± 5% relative humidity and 16:8 h light:dark photoperiod) on a semi-synthetic diet [42]. The *H. armigera* population was established with insects from a laboratory colony maintained continuously in the Universidad Politécnica de Cartagena, Spain. The *S. frugiperda* population was initiated with pupae from a laboratory colony maintained in Honduras and refreshed periodically with insects from southern Mexico. The *M. brassicae* colony was established from pupae gifted by the NERC Centre for Ecology and Hydrology, Oxford.

The H. armigera single nucleopolyhedrovirus (HearSNPV) used in this study was originally isolated from diseased *H. armigera* larvae collected on tomato plants in southern Spain and was named HearSNPV-SP1 [23,41]. The HearMNPV isolate originated from the former USSR [26] and was kindly gifted by Doreen Winstanley (Horticulture Research International, Wellesbourne, Warwick, UK). Virus amplification was performed by feeding *H. armigera* fourth instars with an artificial diet contaminated with OBs of the corresponding virus. OB extraction and purification was performed by filtration through muslin, followed by centrifugation and washing as described previously [41].

The HzAM1 cell line [43] was kindly donated by Robert D. Possee (Centre for Ecology and Hydrology, Wallingford, UK) and was maintained at 28 ± 2 °C using TC100 medium (Lonza, Washington, DC, USA) with the addition of 10% fetal bovine serum (FBS) (Lonza).

### 2.2. Biological Activity of HearSNPV and HearMNPV OBs

To design the coocclusion strategy, first the concentration–mortality response for HearSNPV and HearMNPV OBs was determined by insect bioassay to estimate the 50% lethal concentration (LC_50_), mean time to death (MTD) and OB production (OBs/larva) values. Groups of 30 recently molted *H. armigera* second instar larvae were starved for 12 h and then allowed to drink OB suspensions containing 10% (*w/v*) sucrose, 0.05% (*w/v*) Fluorella Blue food dye and concentrations of OBs that resulted in ~95% to ~5% mortality [44]. For the HearSNPV isolate, these were 5.7 × 10^5^, 1.9 × 10^5^, 6.3 × 10^4^, 2.1 × 10^4^ and 7.0 x 10^3^ OBs/mL, whereas for the HearMNPV isolate these were 1.7 × 10^6^, 5.7 × 10^5^, 1.9 × 10^5^, 6.3 × 10^4^ and 2.1 × 10^4^ OBs/mL. Larvae that ingested the suspension in a 10 min period were individually transferred to 24-well plates with semi-synthetic diet. Control larvae drank a sucrose and dye solution without OBs. Larvae were incubated at 25 ± 1 °C and 70 ± 5% relative humidity until death or pupation. Virus mortality was recorded daily over a 10-day period. The experiment was performed on three occasions (replicates) using different batches of insects. Concentration–mortality data were subjected to Probit analysis using the POLO-PC program [45].

Mean time to death and OB production were determined using the LC_90_ inoculum concentrations, namely 1.7 × 10^5^ and 3.0 × 10^6^ OBs/mL for HearSNPV and HearMNPV isolates, respectively, that resulted in mortalities of 93% and 87%, respectively. Larval mortality was recorded at 8 h intervals in three replicate batches of insects. Moribund individuals, showing clear signs of polyhedrosis disease, were individually transferred to microcentrifuge tubes and incubated at 25 ± 1 °C until death, whereupon insects were immediately frozen at −20 °C. Time–mortality results were subjected to Weibull analysis and the validity of the Weibull model was determined by Kaplan–Meier survival analysis using the GLIM program [46].

To determine OB production, infected cadavers from the mean time to death assay were allowed to thaw, individually homogenized in 1 mL of MilliQ water and triplicate samples of OBs were counted at 400× *g* magnification using a Neubauer improved hemocytometer. OB production values were normalized by logarithmic transformation and were then subjected to analysis of variance (ANOVA) using the SPSS 21.0 program (IBM-SPSS Inc, Chicago, IL, USA).

### 2.3. Superinfection with HearMNPV and HearSNPV at Different Time Intervals

**Figure 1:**
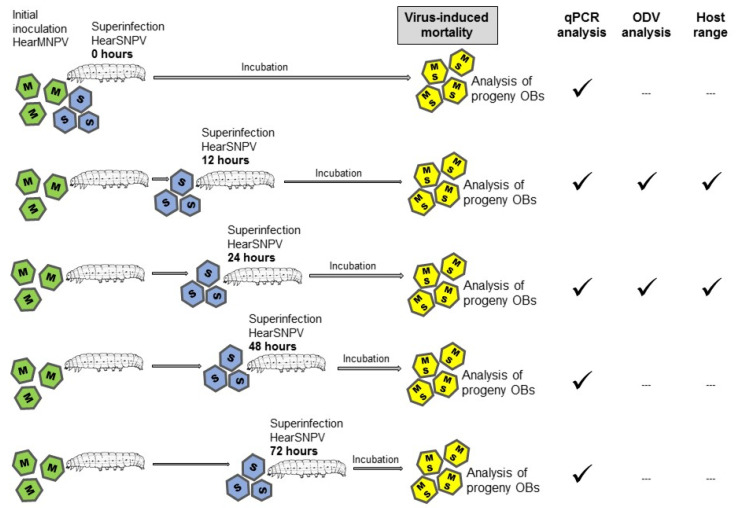
Schematic diagram showing initial inoculation of larvae using HearMNPV inoculum (M, green occlusion bodies [OBs]) followed at intervals of 0, 12, 24, 48 and 72 h by superinfection using HearSNPV inoculum (S, blue OBs). Larvae were then incubated until death and the collection of progeny OBs containing both viruses (M + S, yellow OBs). All the progeny OBs were analyzed by qPCR, but only OBs from the 12 h and 24 h treatments were used for ODV analysis and host range testing.

To obtain OB preparations that contained approximately 50% of HearSNPV and ~50% of HearMNPV genomes, 500 *H. armigera* fifth instar larvae were orally inoculated with the LC_90_ concentration of HearMNPV OBs (previously estimated by bioassay at 2.5 × 10^8^ OBs/mL) using the droplet feeding method (Figure 1). Subsequently, at 0, 12, 24, 48 and 72 h after the initial infection with HearMNPV, a group of 100 of these larvae were orally inoculated with the LC_90_ concentration of HearSNPV OBs (previously estimated at 2.5 × 10^7^ OBs/mL) [41]. As a negative control, a group of 25 larvae were allowed to drink sucrose and dye solution alone without OBs. As positive controls, a group of 25 larvae were inoculated with the LC_90_ concentration of HearMNPV OBs, whereas another group of 25 larvae were inoculated with the LC_90_ concentration of HearSNPV OBs. Larvae were individually transferred to 12-well plates with semi-synthetic diet and incubated at 25 ± 1 °C and 70 ± 5% relative humidity until death (Figure 1). Virus-killed larvae were collected daily, and OBs were purified as described previously [41].

### 2.4. Quantification of HearMNPV and HearSNPV Genomes in OB Samples

The relative prevalence of HearMNPV and HearSNPV genomes in the OB samples obtained following the superinfection procedures was determined by quantitative PCR (qPCR) using specific primers for the HearSNPV and HearMNPV isolates. For HearSNPV, primers were designed in the unique *ha29* gene [31,47]; ha29.1 (5′-CTCGTATCATGCAAAACGCC-3′; nucleotides 25,382 to 25,401 in the HearG4 genome, GenBank accession number AF271059) and ha29.2 (5′-GAATCTGGCTTCGACTGGC-3′; nucleotides 25,443 to 25,461). For HearMNPV, primers targeted ORF63 that was not present in the HearSNPV genome [31,36], and which encodes the *nicotinamide riboside kinase 1* (*nrk1*) gene. This was achieved using primers MNPV.1 (5′-CGTCGACACTCCCAACTGG-3′; nucleotides 58,791 to 58,809 in the HearMNPV genome, GenBank accession number EU730893) and MNPV.2 (5′-CGTTGGACACATGCTGCTG-3′; nucleotides 58,851 to 58,869).

Genomic viral DNA was extracted from samples of 10^7^ OBs by incubation with a solution of 100 µL of 0.5 M Na_2_CO_3_, 50 µL of 10% SDS and 250 µL of MilliQ water at 60 °C during 10 min and then pelleted by centrifugation at 6000× *g* for 5 min. The supernatant was incubated with 25 µL proteinase K (20 mg/mL) at 50 °C during 1 h. Genomic DNA was extracted twice with 500 µL phenol (pH 7.8) followed by treatment with 500 µL chloroform. DNA was precipitated in 10% (*v*/*v*) 3 M sodium acetate (pH 5.2) and 2.5 volumes of 96% ethanol at 12,000× *g* for 10 min and finally washed with 70% ice-cold ethanol. Precipitated DNA was resuspended in 50 µL of 0.1× TE buffer (10 mM Tris, 1 mM EDTA) and stored at 4 °C until use.

All PCR reactions were performed in a total reaction volume of 10 µL comprising 5 µL SsoAdvanced SYBR Green Supermix (Bio-Rad, Berkeley, CA, USA), 0.2 µM final concentration of forward and reverse primers and 1 µL of template DNA. Non-template controls (NTCs), standard curves (30 to 1.9 × 10–3 ng/µL of serial five-fold dilutions of template DNA), and samples were analyzed in duplicate. All qPCR reactions were performed in a CFX96 Touch Real-Time PCR Detection System (Bio-Rad, Hercules, CA, USA). The program used was: 2 min 30 sec at 95 °C; 45 cycles of 98 °C for 15 sec and 60 °C for 30 sec, followed by a melting curve (60–95 °C). Data were analyzed using Bio-Rad CFX Manager software (Bio-Rad, Hercules, CA, USA).

### 2.5. Analysis of Mixed-Virus ODVs

First, the presence of HearSNPV genomes within HearMNPV multinucleocapsid ODVs was determined by PCR on samples the ODV bands extracted from sucrose gradients. For this, ODVs were released from samples of 10^9^ OBs from the superinfection preparations at different time intervals (T0, T12, T24, T48 and T72) by incubation with 0.1 M Na_2_CO_3_ for 30 min at 28 °C. Debris was removed by low speed centrifugation (2500× *g*) for 5 min. The ODV containing supernatant was subjected to density equilibrium centrifugation at 30,000× *g* during 1 h on a 30–70% (*w*/*w*) continuous sucrose gradient. The resulting banding patterns were inspected and photographed. The upper band, comprising ODVs with a single nucleocapsid, was extracted by puncturing the tube at the height of the band with a syringe needle and transferred to a 2 mL sterile tube. In addition, the two or three lower bands, comprising ODVs with several nucleocapsids, were extracted and pooled to produce a ‘multinucleocapsid ODV sample’. A 100 µL volume of each ODV sample was treated with 3 µL proteinase K (20 mg/mL) at 50 °C during 30 min and boiled at 100 °C for 10 min, and then used as the template for a qPCR analysis as described in Section 2.4.

Second, to ensure that the results were not affected by adhesion between HearSNPV ODVs and HearMNPV ODVs, or contamination during sample collection, a control experiment was performed in which four samples (replicates) of 5 × 10^9^ HearSNPV OBs were mixed with 5 × 10^9^ HearMNPV OBs in 1 mL of distilled water. The mixture was subjected to alkaline lysis and continuous sucrose gradient centrifugation as described for the superinfection samples. The upper and lower bands of ODVs were collected and subjected to qPCR analysis in triplicate to estimate the abundance of HearSNPV genomes using the primers for the *ha29* gene (see Section 2.4). The results were normally distributed but differed in variances (heteroscedasticity) and were therefore analyzed by Welch’s analysis of variance in the R-based Jamovi package [48].

Third, co-envelopment of HearSNPV and HearMNPV genomes within the same ODV was verified by end-point dilution assays. ODVs from samples of 10^8^ OBs produced in the T12 and T24 superinfection treatments (Section 2.3) were released by incubation with 0.1 M Na_2_CO_3_ for 30 min at 28 °C, then centrifuged at 2500× *g* during 5 min, and ODVs containing supernatants were serially diluted (10^−1^ to 10^−5^) in TC100 medium (Lonza) supplemented with 1% antibiotic mixture of penicillin and streptomycin (Lonza). A 100 µL volume of each dilution was mixed with 900 µL of cell suspension (2 × 10^5^ HzAM1 cells/mL). A 100 µL volume of each ODV-cell suspension was added to each well of a 96 well plate, leaving the last well as a negative control, with cells but without virus. The assay was replicated three times. Plates were incubated at 28 °C. After 7 days, all wells were examined to determine the presence of infected cells with OBs in the nucleus. Plates from the dilution that resulted in less than 15% of infected wells were examined, individual infected wells were extracted using a sterile Pasteur pipette and used as template in PCR and qPCR amplifications, after treatment with 5 µL proteinase K (20 mg/mL) at 50 °C during 30 min followed by 10 min at 100 °C. PCR amplification was performed using Taq DNA polymerase (Bioline, London, UK) and specific primers for the HearSNPV or HearMNPV genomes. For HearSNPV amplification, the specific primers were also designed in the unique *ha29* gene [31,47]; ha29.3 (5′-ATCGCACCATACCATGTATC-3´; nucleotides 25,251 to 25,270 in the HearG4 genome) and ha29.4 (5´-ATATCGCGATAACTAGTGGC-3´; nucleotides 25,639 to 25,658), whereas for HearMNPV, the primers targeted the ORF2, that encodes the viral capsid associated protein, which is absent in HearSNPV [31,36]. These primers were MNPV.3 (5′-GGTAAGAAAGATCCAGACG-3′; nucleotides 1529 to 1557 in the HearMNPV genome) and MNPV.4 (5′-CGTCCAAAATTGCTATTCTTG-3′; nucleotides 2082 to 2102). The resulting PCR products were subjected to electrophoresis in 1% agarose gel containing 0.25 µg/mL ethidium bromide in TAE buffer (40 mM Tris, 20 mM acetic acid, 1 mM EDTA) at 80 V for 1 h and photographed on a GeneSnap (Syngene, Cambridge, UK) UV-transilluminator. DNA fragment sizes were estimated by comparison to a standard molecular weight marker (100 bp Nippon Genetics Europe GmbH, Düren, Germany). The qPCR analysis was performed as described in Section 2.4.

### 2.6. Biological Activity of Cooccluded Mixed-Virus OBs

The biological activity of the OBs obtained following inoculation of larvae with HearMNPV and HearSNPV at intervals of 12 and 24 h (T12 and T24 samples) was determined by droplet feeding bioassays in *H. armigera*, *M. brassicae* and *S. frugiperda* larvae. All these species are susceptible to infection by HearMNPV, whereas HearSNPV is only infective for *H. armigera*. In addition, pure HearMNPV OBs and HearSNPV OBs were mixed in a 1:1 ratio (50% HearSNPV OBs + 50% HearMNPV OBs) and included as a reference treatment.

The LC_50_ values were estimated using five different OB concentrations as described in Section 2.2. OB concentrations used to inoculate *H. armigera* larvae were 5.7 × 10^5^, 1.9 × 10^5^, 6.3 × 10^4^, 2.1 × 10^4^ and 7.0 × 10^3^ OBs/mL for the inocula comprising HearSNPV, T12, T24 and the OB mixture 50% HearSNPV OBs + 50% HearMNPV OBs, whereas the concentrations used to inoculate *H. armigera* larvae with HearMNPV OBs alone were: 1.7 × 10^6^, 5.7 × 10^5^, 1.9 × 10^5^, 6.3 × 10^4^ and 2.1 × 10^4^ OBs/mL. In bioassays involving *S. frugiperda*, OB concentrations used for inocula comprising HearSNPV, T12, T24, and the OB mixture 50% HearSNPV OBs + 50% HearMNPV OBs, were 1.5 × 10^9^, 3.1 × 10^8^, 6.2 × 10^7^, 1.2 × 10^7^ and 2.5 × 10^6^ OBs/mL, and for HearMNPV OBs alone were 3.1 × 10^8^, 6.2 × 10^7^, 1.2 × 10^7^, 2.5 × 10^6^ and 5.0 × 10^5^ OBs/mL. Finally, in *M. brassicae* the same range of inoculum concentrations was used in all treatments: 3.1 × 10^6^, 6.2 × 10^5^, 1.2 × 10^5^, 2.5 × 10^4^ and 5.0 × 10^3^ OBs/mL. All bioassays were performed on three batches of insects (replicates). Interactions between viruses in larvae inoculated in the T12, T24 and the 50% HearSNPV OBs + 50% HearMNPV OBs treatments were evaluated using the method of Tabashnik [49]. In this method, the expected LC_50_ value of the mixture (LC_50*m*_) is estimated from the relative proportions (*r*_HearSNPV_, *r*_HearMNPV_) of HearSNPV and HearMNPV and the LC_50_ values of each component using the equation LC_50*m*_ = [*r*_HearSNPV_/LC_50HearSNPV_ + *r*_HearMNPV_/LC_50HearMNPV_]^−1^ [49]. As such, a lower LC_50*m*_ value than that predicted by Tabashnik’s formula would indicate a synergistic interaction, whereas a higher value would indicate an antagonistic interaction.

### 2.7. Stability of Mixed-Virus Preparations during Serial Passage in Larvae

Larvae that died of lethal polyhedrosis at the highest inoculum concentration in the T12, T24 and 50% HearSNPV OBs + 50% HearMNPV OBs mixture in bioassays (Section 2.6) were collected and pooled within each treatment. These OBs, representing the total OB production of the infected group of larvae, were considered to be passage one (P1) OBs and were used as inoculum to infect a group of 24 *H. armigera* second instars. Each virus population was subjected to four additional passage steps (P2, P3, P4 and P5). Additionally, as positive controls, groups of 24 s instars of *H. armigera*, *M. brassicae* and *S. frugiperda* larvae were inoculated with an LC_90_ concentration of OBs from the superinfection preparations involving the HearSNPV (T0) and HearMNPV (T48 and T72) treatments. Specifically, *H. armigera* larvae were inoculated with 5 × 10^5^, 2 × 10^6^ and 2 × 10^6^ OBs/mL of the T0, T48 and T72 samples, respectively. Larvae of *S. frugiperda* were inoculated with 2 × 10^9^ OBs/mL of the T0 sample, or 3 × 10^8^ OBs/mL for the T48 and T72 samples. Finally, *M. brassicae* larvae were inoculated with 2 × 10^9^, 3 × 10^6^ and 3 × 10^6^ OBs/mL of the T0, T48 and T72 samples, respectively. Inoculated larvae were reared individually until death. Virus-killed insects were collected in groups and the OBs were designated as passage one (P1), but these samples (T0, T48, T72) were not subjected to additional steps of serial passage. The entire experiment was performed three times (replicates). Viral DNA was extracted from purified OBs at each step of passage and was subjected to PCR and qPCR amplifications as described in Section 2.4, to determine the relative prevalence of HearSNPV and HearMNPV genomes in each OB sample at each passage.

## 3. Results

### 3.1. Insecticidal Characteristics of HearSNPV and HearMNPV OBs

HearSNPV OBs (LC_50_ = 2.9 × 10^4^ OBs/mL) were 6.2-fold more pathogenic than HearMNPV OBs (LC_50_ = 1.8 × 10^5^ OBs/mL) in *H. armigera* second instars (Table 1). The *H. armigera* larvae treated with HearSNPV died an average of 130 h after inoculation, while those treated with HearMNPV died ~13 h later. None of the control larvae died from polyhedrosis disease.

The mean number of OBs produced in each larva averaged 1.1 × 10^8^ OBs/larva in HearSNPV-killed insects but was almost ~50% lower in HearMNPV-killed insects (Tukey, *p* < 0.05) (Table 1).

### 3.2. Relative Prevalence of HearSNPV and HearMNPV Genomes in Mixtures

**Figure 2:**
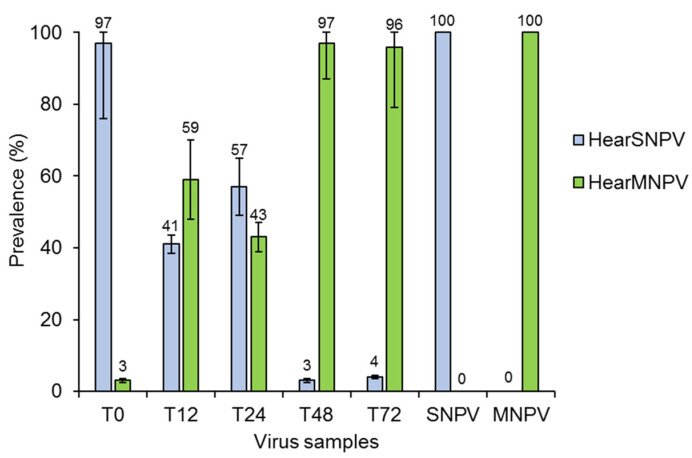
Relative prevalence (%) of HearSNPV and HearMNPV genomes determined by qPCR of progeny occlusion bodies obtained after initial infection of *Helicoverpa armigera* larvae with an LC_90_ concentration of HearMNPV followed at intervals of 0 h (T0), 12 h (T12), 24 h (T24), 48 h (T48) and 72 h (T72) by superinfection with an LC_90_ concentration of HearSNPV occlusion bodies. Vertical lines indicate asymmetrical standard error. Values above bars indicate means.

When *H. armigera* larvae were inoculated simultaneously with HearMNPV and HearSNPV (T0), qPCR analysis indicated that HearSNPV was the most frequent virus (96.8%) in progeny OBs produced in virus-killed larvae (Figure 2). However, the prevalence of both viruses in progeny OBs was similar when HearSNPV OBs were ingested by the larvae 12 h (41.1% of HearSNPV, 58.9% of HearMNPV) or 24 h (57.3% of HearSNPV, 42.7% of HearMNPV) later than HearMNPV OBs. By contrast, HearMNPV was the dominant component in the OBs produced in larvae inoculated with HearSNPV OBs at 48 h (96.8%) or 72 h (95.6%) hours after HearMNPV inoculation (Figure 2). When genome copy number values were calculated it was clear that OBs from treatment T0 had a 1.5 logarithm lower concentration of HearMNPV compared to HearSNPV, whereas the number of genome copies of the multinucleocapsid virus was 1.3–1.4 logarithms higher than that of HearSNPV in the progeny OBs from the T48 and T72 treatments (Supplementary Figure S1). The control larvae inoculated with each virus separately showed no signs of cross-contamination.

### 3.3. HearSNPV and HearMNPV Genomes Are Present within the Same ODV

**Figure 3:**
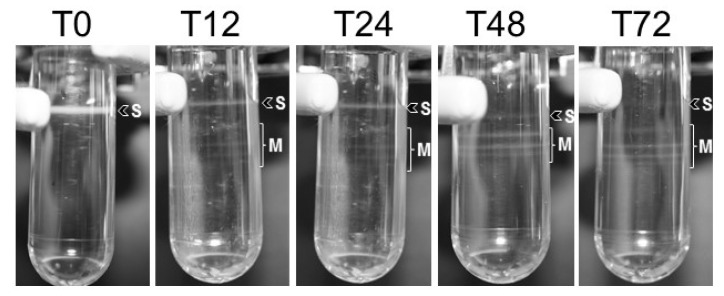
ODV banding patterns of OB samples (T0–T72) after continuous sucrose gradient separation of ODVs released from 10^9^ OBs. Arrowhead (S) indicates band of single nucleocapsid ODVs, bracket (M) indicates bands comprising multinucleocapsid ODVs that were pooled for analysis.

OBs recovered from virus-killed larvae that ingested both viruses simultaneously (HearSNPV and HearMNPV) mainly consisted of single nucleocapsid ODVs as they migrated as a single band when subjected to centrifugation (T0 in Figure 3). Other bands were not seen in the sucrose gradient of the T0 treatment. OB samples obtained from larvae inoculated first with HearMNPV and 12, 24, 48 and 72 h later with HearSNPV (T12, T24, T48 and T72 samples) were composed of ODVs with multiple nucleocapsids, as multiple bands were clearly visible (Figure 3).

**Figure 4:**
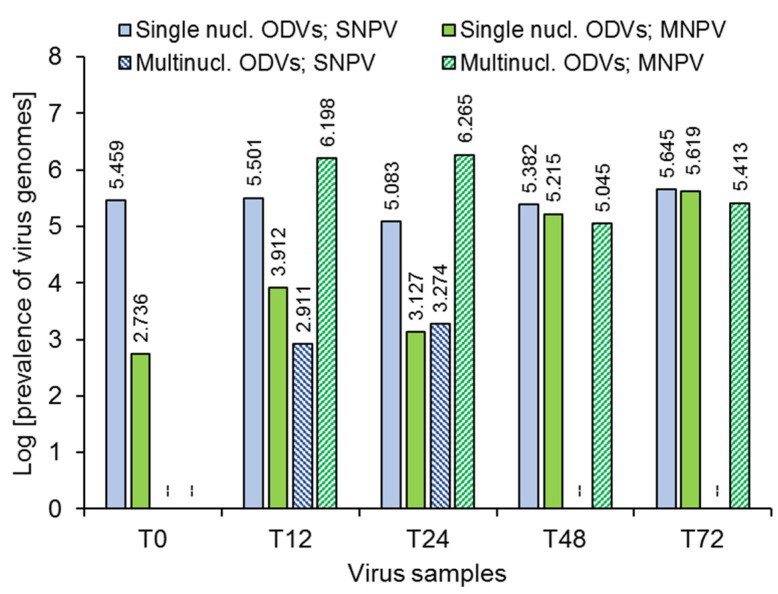
Results of qPCR quantification of genome copy number for HearSNPV and HearMNPV in single nucleocapsid ODVs (solid-colored columns) and multinucleocapsid ODVs (hatched columns) obtained from OBs collected from the T0, T12, T24, T48 and T72 treatments. Values above bars indicate logarithm of the prevalence of virus genomes. Dashed lines indicate that no sample was present or could be collected.

Quantitative PCR presented a clear picture of the composition of ODV bands (Table 2, Figure 4). Analysis of single nucleocapsid ODVs revealed that simultaneous inoculation of larvae with both viruses (T0) resulted in a 99.78% prevalence of HearSNPV genomes and a 0.22% prevalence of HearMNPV genomes, whereas multinucleocapsid ODVs were not visible in this sample and could not be analyzed. However, as the interval between first (HearMNPV) and second (HearSNPV) inoculation increased the prevalence of the former increased while the latter decreased in single nucleocapsid ODVs, to a maximum of 47.73% of HearSNPV and 52.27% HearMNPV genomes in the T72 treatment. In contrast, analysis of multinucleocapsid ODVs indicated the presence of 0.47–0.88% of HearSNPV genomes in the T12 and T24 samples, whereas in later time point samples, only HearMNPV genomes could be detected (Table 2).

Analysis of ODV composition in terms of genome copy numbers revealed a difference of 2.7, 1.6 and 2.0 logarithms in favor of HearSNPV genomes over HearMNPV genomes present in single nucleocapsid ODVs at T0, T12 and T24, respectively, whereas numbers of genome copies of the different viruses in single nucleocapsid ODVs were similar in T48 and T72 samples (Figure 4). Analysis of the pooled samples of multinucleocapsid ODVs revealed a 3.3 and 3.0 logarithm difference in favor of HearMNPV genomes in the T12 and T24 treatments, respectively. However, between ~800 and ~1800 HearSNPV genomes were detected in the multinucleocapsid ODV samples from the T12 and T24 treatments, respectively, but were not detected in the T48 and T72 samples of multinucleocapsid ODVs (Figure 4).

These findings suggest that HearSNPV was capable of markedly suppressing coinfection and/or replication by HearMNPV in larvae infected with HearMNPV up to 24 h previously. However, when the interval between inoculations was 48 h or greater, HearMNPV-infected cells were capable of absolute exclusion of superinfection by HearSNPV, resulting in the complete absence of HearSNPV in multinucleocapsid ODVs from 48 h onwards.

As a control experiment, to test possible contamination of multinucleocapsid (lower bands) samples by HearSNPV through adhesion of ODVs or accidental contamination during band extraction from sucrose gradients, OBs of both viruses were mixed, ODVs were separated by centrifugation and qPCR analysis was performed on upper and lower band samples using primers targeted at the unique *ha29* gene of HearSNPV. The estimated copy number of HearSNPV genomes differed significantly between upper and lower band samples (Welch’s F = 606.6, d.f. = 1, 3.238, *p* < 0.001). Estimates from the upper band samples averaged 10^6.372^ ± 10^0.396^ (mean ± SD) equivalent to 2.36 × 10^6^ copies, with a mean (±SD) Cq value of 11.94 ± 0.99 that varied little among the four replicate samples (Table 3). In contrast, the estimated HearSNPV copy number from the lower bands was 10^1.400^ ± 10^0.079^ (equivalent to 25.12 copies) with a mean Cq value of 25.95 ± 0.20; values that were similar to those obtained from the amplification of the control samples of multinucleocapsid virus HearMNPV (mean ± SD: 10^1.431^ ± 10^0.001^, equivalent to 26.96 copies, with a mean Cq value of 24.30 ± 0.03). It was clear, therefore, that the experimental results on qPCR analysis and end-point dilution were unlikely to have been affected by adhesion of single and multinucleocapsid ODVs or accidental contamination of upper and lower band samples during band extraction.

**Figure 5:**
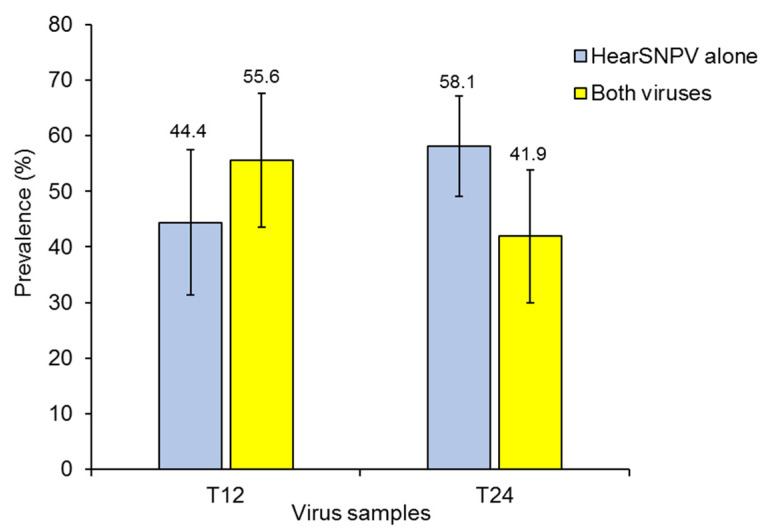
Prevalence (%) of wells positive for HearSNPV alone or mixtures of HearSNPV and HearMNPV (both viruses). PCR was performed using virus-specific primers on the 27 and 31 infected wells that were observed following end-point dilution of ODVs released from the T12 and T24 treatment OBs, respectively. Vertical lines indicate standard error. Values above bars indicate means.

End point dilution assays were performed on samples of ODVs from the T12 and T24 treatments. A dilution of 10^−3^ resulted in ~90% of uninfected wells, which reflected the situation in which ~10% of the wells were infected by a single ODV and 1% or less of wells were infected by two or more ODVs, following the Poisson probability distribution (Table 4). For example, the estimated probability of infection by two ODVs ranged from 0.41 to 0.78% in the T12 treatment assay, and from 0.32–1.09% in the T24 treatment assay (Table 4). Of a total of 27 wells analyzed by PCR in the three replicate samples from the T12 treatment, 12 wells were infected HearSNPV alone (44%) and 15 comprised a mixture of both viruses (56%) (Figure 5). Similarly, of the 31 wells analyzed in the T24 treatment, 18 comprised HearSNPV alone (58%) and 13 comprised a mixture of both viruses (42%) (Figure 5). The prevalence of wells with single and mixed-virus infections was similar in the T12 and T24 treatments (Fisher’s exact test *p* = 0.429). However, in those wells where both viruses were detected, qPCR analysis indicated that 88.4 ± 18.8% and 69.4 ± 39.3% of the viral DNA was HearSNPV in T12 and T24 samples, respectively. As only 0.47–0.88% of HearSNPV genomes were present in multinucleocapsid ODVs comprised (Table 2), this difference is probably because HearSNPV is more amenable to replication in HzAM1 cells than HearMNPV, i.e., ODVs that contained HearSNPV genomes were more efficient at infection and replication in the end-point dilution assay. It was clear, however, that HearSNPV nucleocapsids were present in a fraction of the ODVs containing HearMNPV nucleocapsids.

### 3.4. Biological Activity of Cooccluded Mixtures

Mixed-virus preparations that comprised near equal proportions of each virus (T12 and T24 samples) were subjected to bioassays to determine OB pathogenicity, speed of kill, OB production, and host range characteristics compared to each virus alone. For this, bioassays were performed with three different host species. The homologous host *H. armigera* is more susceptible to HearSNPV than to HearMNPV, whereas *S. frugiperda* and *M. brassicae* are only permissive to HearMNPV [22,35,37,38].

For *H. armigera* larvae, the LC_50_ values of the T12 treatment OBs (2.2 × 10^4^ OBs/mL), T24 OBs (3.5 × 10^4^ OBs/mL), and the OB mixture comprising 50% HearSNPV OBs + 50% HearMNPV OBs (3.0 × 10^4^ OBs/mL) did not differ significantly from the LC_50_ value of HearSNPV OBs alone (1.5 × 10^4^ OBs/mL), although they were 2.9-fold to 4.5-fold more pathogenic than HearMNPV OBs alone (1.0 × 10^5^ OBs/mL) (Table 5). When larvae of *S. frugiperda* and *M. brassicae* ingested the mixed-virus preparations T12 and T24, or the 50% HearSNPV OBs + 50% HearMNPV OBs mixture, the LC_50_ values were similar to one another, both in the case of *S. frugiperda* larvae (3.6 × 10^7^–4.2 × 10^7^ OBs/mL) and for *M. brassicae* (3.9 × 10^5^–5.5 × 10^5^ OBs/mL). However, these LC_50_ values were all approximately two-fold lower than the LC_50_ values of HearMNPV OBs in *S. frugiperda* (2.0 × 10^7^ OBs/mL) and *M. brassicae* (2.0 × 10^5^ OBs/mL) (Table 5).

The expected LC_50_ values of these mixtures against *H. armigera*, *S. frugiperda* and *M. brassicae* larvae calculated according to Tabashnik’s equation (Table 5) were clearly within the 95% confidence limits of each of these LC_50_ values, indicating the absence of synergistic or antagonistic interactions between HearSNPV and HearMNPV in the OB pathogenicity of mixed-virus preparations in each of the different host species.

### 3.5. Host Range and Stability of Mixed-Virus Preparations in Serial Passage

Quantitative PCR analysis of the progeny OBs that resulted from a single inoculation step (P1) in *H. armigera*, revealed that the prevalence of HearSNPV genomes increased markedly in all mixed-virus samples (T0–T72) compared to the initial inocula (P0) (Table 6). When these preparations were used to inoculate *S. frugiperda* or *M. brassicae* larvae, very low levels of HearSNPV genomes were detected in the P1 OBs which did not differ clearly among the cooccluded preparations or compared to the mixture of 50% HearSNPV OBs + 50% HearMNPV OBs.

The stability of the cooccluded mixture of viruses in the T12 and T24 preparations and the 50% HearSNPV + 50% HearMNPV mixture was determined over five successive passages in vivo. These experiments were performed in parallel in *H. armigera*, *S. frugiperda*, and *M. brassicae* larvae.

In *H. armigera* larvae, the relative prevalence of HearSNPV increased markedly after a single pass from 41.0% (P0) to 99.9% (P1) in the T12 preparation and from 57.3% to 96.5% in the T24 preparation, which was similar to the increase from 50.0% to 97.8% observed in the 50% HearSNPV OBs + 50% HearMNPV OBs mixture (Table 6). The presence of HearMNPV genomes was not detected in any of the OBs samples from subsequent passages (P2–P5) indicating that HearMNPV had been completely displaced by HearSNPV (Table 6).

The opposite trend was observed in heterologous hosts. The prevalence of HearSNPV genomes decreased markedly after one passage in *S. frugiperda* or *M. brassicae* larvae and remained extremely low thereafter, indicating that HearSNPV had been displaced by HearMNPV in the heterologous hosts (Table 6). Interestingly, HearSNPV was not completely eliminated during serial passage of the T12 and T24 preparations in the heterologous hosts and persisted at very low prevalence (0.00001–0.00006%) in *S. frugiperda* and a slightly higher prevalence (0.00018–0.01987%) in *M. brassicae* larvae in OB samples from passage steps P2–P5. This trend was also observed in the 50% HearSNPV OBs + 50% HearMNPV OBs mixture in both heterologous species, in which HearSNPV genomes presumably became cooccluded with HearMNPV genomes during the P1 coinfection step and in all subsequent passage steps (Table 6).

## 4. Discussion

This study demonstrates for the first time that by coinfecting *H. armigera* larvae with the homologous single nucleocapsid (HearSNPV) and multinucleocapsid (HearMNPV) NPVs, under certain conditions, both viruses can coinfect host cells resulting in coocclusion of the viruses in progeny OBs. Moreover, it was possible to obtain a fraction of the ODVs comprising co-enveloped genomes of both viruses. Previous studies have shown that individual host cells can be infected by multiple genotypes of alphabaculovirus [13,16]. Furthermore, individual insect cells can be coinfected with different alphabaculovirus species, provided that coinfection occurs within a given time window [15]. For example, when Autographa californica multiple nucleopolyhedrovirus (AcMNPV) and Spodoptera frugiperda multiple nucleopolyhedrovirus (SfMNPV) were used to sequentially coinfect Sf9 cells, productive infection by the second virus was only possible within ~16 h following infection by the first virus. After this time a cytoskeletal block prevents productive coinfection by the second virus [21].

When *H. armigera* larvae were simultaneously coinfected with HearSNPV and the less pathogenic and less virulent multinucleocapsid HearMNPV, progeny OBs contained mostly HearSNPV genomes (97%), probably due to the higher capacity for replication of HearSNPV in *H. armigera*. However, at an organismal level, when larvae of *H. armigera* were first infected by the less pathogenic and less virulent HearMNPV, no absolute exclusion to subsequent superinfection by HearSNPV was observed, although the prevalence of HearSNPV progeny was markedly reduced when HearSNPV inoculation was delayed by more than 24 h. The low prevalence of HearSNPV at later time points likely reflects the reduced number of cells that had not already been infected by HearMNPV at later times post-inoculation.

Partial superinfection exclusion was observed previously in *S. frugiperda* larvae that were initially inoculated with SfMNPV OBs and subsequently inoculated with AcMNPV OBs. However, when *S. frugiperda* larvae were first inoculated with the less pathogenic virus, AcMNPV, and subsequently inoculated with the homologous virus, SfMNPV, total superinfection exclusion was observed [50]. Therefore, each host-pathogen system is likely to require prior calibration when constructing precise cooccluded mixtures of viruses.

When larvae were inoculated with HearSNPV 12 or 24 h later than HearMNPV, similar proportions of both viruses were obtained in OBs from coinfected larvae, suggesting that this time interval was sufficient to offset the replication disadvantage of HearMNPV in *H. armigera*. The ODVs released from these OBs separated into several bands by ultracentrifugation, each corresponding to ODVs with a specific number of nucleocapsids. HearSNPV was detected by PCR in multinucleocapsid ODVs, suggesting that a modest number of HearSNPV nucleocapsids were enveloped within HearMNPV ODVs. This was confirmed by end point dilution assays.

Both HearSNPV and HearMNPV genomes were detected in wells infected by a single ODV indicating co-envelopment of the viruses. An alternative explanation, in which single and multinucleocapsid ODVs adhere to one another during the cell inoculation procedure, was shown to be highly unlikely as qPCR analysis of HearSNPV genomes in mixtures of HearSNPV and HearMNPV ODVs separated by centrifugation did not exceed negative control levels and threshold Cq values were extremely high (Cq ~25). A previous study also reported adhesion between ODVs of two different NPVs to be a rare event [15].

The estimates of the prevalence of co-envelopment of HearSNPV and HearMNPV genomes varied from <1% by qPCR analysis of ODV bands to 41.9–55.6% by PCR analysis of infected wells in the end-point dilution assay (Figure 5). None of the wells were infected by HearMNPV alone. This disparity is likely to have arisen through a combination of two factors (i) HearMNPV is less amenable to replication in cell culture than HearSNPV and, (ii) ODVs produced in cells infected by both viruses will have acquired a pseudotype reflecting the parental mixture of viruses, i.e., ODVs produced in coinfected cells will have acquired an infectious phenotype similar to that of HearSNPV and will be more infectious in cell culture than ODVs produced in cells infected by HearMNPV alone. The sharing of the protein pool derived from the transcription of both parental viruses, resulting in virion pseudotyping, is an example of *cis*-acting factors that modulate virion infectivity [51]. A similar approach was recently used to explore the effect of heterologous *per os* infection factors on the oral infectivity of HearSNPV ODVs [52].

We then explored the effects of coocclusion of HearSNPV and HearMNPV on the pathogenicity of mixed-virus OBs. The T12 and T24 OB mixtures were equally pathogenic as the mixture of 50% HearSNPV OBs + 50% HearMNPV OBs against all three host species. Each virus acted independently, indicating that cooccluded viruses and the mixture of OBs were transmitted in a similar manner. Comparable results were observed in *S. frugiperda* and *S. exigua* larvae after inoculation with cooccluded preparations and mixtures of AcMNPV OBs + SfMNPV OBs; both viruses were transmitted independently, with no apparent interaction in terms of OB pathogenicity [15]. In contrast, the cooccluded mixtures exhibited a lower pathogenicity than HearSNPV OBs alone against *H. armigera*. The cooccluded mixtures were also slightly less pathogenic than HearMNPV against *S. frugiperda* and *M. brassicae*, which are both semi-permissible to HearMNPV (Table 4). This lower pathogenicity may be because T12 and T24 OB samples comprised ~50% of HearSNPV and ~50% of HearMNPV genomes, which reduced the presence of the more pathogenic virus by ~50% in homologous and heterologous hosts, respectively.

The coocclusion of mixed-virus preparations could have clear implications in insect control programs, as these mixtures provide a functional increase in the host range of NPV-based insecticides. As such, cooccluded mixtures could form the basis for pest control products targeted at two or more insects for a given crop [50]. For example, although *H. armigera* is the most important pest of tomato crops in Spain [53], *M. brassicae* is also present in this crop [54]. In addition, *M. brassicae* is a major pest of cabbage in Europe [55], and *H. armigera* may also cause damage in this and other brassica crops [56]. Similarly, *S. frugiperda* is now an important pest in the Americas, Africa and Asia, where it attacks maize, rice, soybean and cotton [57] and *H. armigera* has been introduced to South America where it is causing significant damage in soybean and cotton crops [1]. In these cases, applications of cooccluded mixtures of HearSNPV and HearMNPV might allow the simultaneous control of both pests, although this required verification in careful field testing. Moreover, other researchers are exploring the application of virus coocclusion technology for the control of complexes of lepidopteran pests in certain crops [58,59]. Commercial producers have also addressed this issue by mixing OBs of different viruses to create products capable of controlling complexes of pests [60].

The cooccluded virus mixtures were unstable and the relative proportion of each virus varied markedly depending on the host species in which the mixture replicated. When mixtures were passaged in *H. armigera* larvae the prevalence of HearMNPV genomes decreased from ~50% to less than 4%, and then disappeared after two passages. Conversely, when mixtures were passaged in *S. frugiperda* and *M. brassicae*, the relative proportion of HearMNPV increased form ~50% to 99.9% in just one passage, corroborating the restricted host range of HearSNPV. Serial passage has been used as a tool to increase the insecticidal activity of alpha- and betabaculoviruses in semi-permissive hosts [61,62,63,64], although not all of these studies had molecular tools available to verify the identity of progeny OBs and rule out the possible activation of a latent infection in the heterologous host. A previous study of HearMNPV in two heterologous hosts (*S. exigua* and *S. littoralis*) indicated that this virus did not undergo marked changes in OB pathogenicity characteristics in either host during serial passage, although significant changes in speed of kill were detected in some lineages [39]. By contrast, serial passage at high inoculum concentrations was used to examine how the diversity of an indicator gene (*dnapol*) varied during the process of adaptation of a HearSNPV isolate in individual insects of the homologous host [65], or how changes in the relative prevalence of cooccluded genotypes affected the insecticidal phenotype in HearSNPV [66] and SfMNPV [67].

Serial passage experiments have shown that these viruses undergo genetic and phenotypic changes due to genetic bottlenecks and genetic drift [63,68]. Adaptation to a heterologous host can involve genetic diversification, including alterations in the abundance of certain genotypic variants or the emergence of new genotypes due to recombination events [63,69]. Replication in a particular host also affects virion composition as both budded virus and ODVs can be contaminated by host-specific proteins [70]. Spontaneous generation of genetic diversity has also been reported in nucleopolyhedrovirus variants, including HearSNPV, especially at low inoculum doses [18,71]. However, the high inoculum doses used in the present study would likely have obfuscated the spontaneous generation of variability, although we did not address this issue in the present study.

The insect midgut is a key site in the selection of viruses [65,72,73], especially as species specific interactions between host proteins and complexes of *per os* infection factors on the surface of ODVs are key to the initial entry of viral nucleocapsids into midgut epithelial cells [74,75]. Processes such as host innate immune defenses, variation in budded virus dispersal to other host tissues, or the presence of defective genotypes in the natural virus isolates are also likely to determine the success of infections in homologous and heterologous hosts [76,77]. In this sense, a detailed comparison of the single and multinucleocapsid packaging strategies revealed that Helicoverpa zea SNPV established an infection in midgut epithelial cells faster, in greater numbers, and spread more rapidly in systemic infection than an equivalent dose of the multinucleocapsid AcMNPV. However, AcMNPV was more efficient at establishing infection on a per virion basis and was capable of rapid repackaging of nucleocapsids to produce budded virions in the absence of a complete replication cycle, allowing systemic infection to proceed despite increased sloughing of midgut cells [78].

## 5. Conclusions

This study demonstrates for the first time that by coinfecting *H. armigera* larvae with the homologous single nucleocapsid (HearSNPV) and multinucleocapsid (HearMNPV) laboratory-controlled mixtures of HearSNPV and HearMNPV were cooccluded to produce mixed-virus OBs. A fraction of HearSNPV nucleocapsids were enveloped within multinucleocapsid ODVs. The composition of the cooccluded mixture could be modulated by altering the time interval between inoculations, but the mixture was unstable during serial passage. Virion pseudotyping in coinfected cells was likely responsible for the high infectivity of mixed-virus ODVs in cell culture conditions. This strategy may be applied to the production of virus-based insecticides that have the capacity to control two or more lepidopteran pests in a particular crop, although this requires validation through field testing.

## Figures and Tables

**Table 1:** Probit analysis of concentration–mortality responses used to estimate LC_50_ and relative potency values. Mean time to death (MTD) values and OB production/larva of HearMNPV and HearSNPV occlusion bodies (OBs) in *Helicoverpa armigera* second instar larvae.

Virus	LC_50_ (OBs/mL)	Potency	Range of 95% C.I.	MTD (h)	Range of 95% C.I.	Mean OB Production (±SE) (×10^7^)
HearMNPV	1.8 × 10^5^ a	1	-	143.4a	140.4–146.5	6.1 ± 1.2 a
HearSNPV	2.9 × 10^4^ b	6.2	4.0–10.3	130.0b	127.3–132.7	11 ± 2.4 b

A test for non-parallelism of probit regression slopes was significant (χ^2^ = 5.94, df = 1, *p* = 0.015). Relative potency values were calculated as the ratio of LC_50_ values relative to that of HearMNPV. The mean time to death (MTD) values were estimated by Weibull analysis. Values followed by different letters differ significantly (MTD, *t*-test, *p* < 0.05; OB production ANOVA, Tukey *p* < 0.05).

**Table 2:** Relative prevalence (%) of HearSNPV and HearMNPV genomes in single nucleocapsid and multinucleocapsid ODVs obtained from OBs collected from the T0, T12, T24, T48 and T72 treatments determined by qPCR using virus-specific primers.

	Single Nucleocapsid ODVs		Multinucleocapsid ODVs ^1^	
Sample	HearSNPV	HearMNPV	HearSNPV	HearMNPV
T0	99.78	0.22	-	-
T12	97.09	2.91	0.47	99.53
T24	98.73	1.27	0.88	99.12
T48	55.77	44.23	0.00	100.00
T72	47.73	52.27	0.00	100.00

^1^ Multinucleocapsid ODVs were not observed following centrifugation of T0 samples.

**Table 3:** Quantitative PCR analysis of single nucleocapsid (upper) and multinucleocapsid (lower) bands of occlusion derived virions released from a mixture of HearSNPV OBs and HearMNPV OBs. The logarithm of HearSNPV genome copy number and quantification cycle (Cq) values were determined using primers targeted at the unique *ha29* gene.

Sample	Replicate	Log[Copy Number] (mean ± SD)	Cq Value (mean ± SD)
Upper band	Replicate 1	6.686 ± 0.096	11.16 ± 0.21
	Replicate 2	6.743 ± 0.050	11.01 ± 0.13
	Replicate 3	6.019 ± 0.027	12.83 ± 0.07
	Replicate 4	6.041 ± 0.090	12.77 ± 0.23
	Mean ± SD	6.372 ± 0.396	11.94 ± 0.99
Lower bands	Replicate 1	1.355 ± 0.062	24.49 ± 0.16
	Replicate 2	1.462 ± 0.037	24.22 ± 0.09
	Replicate 3	1.312 ± 0.049	24.60 ± 0.12
	Replicate 4	1.471 ± 0.175	24.20 ± 0.44
	Mean ± SD	1.400 ± 0.079	25.95 ± 0.20

Values indicate means (± SD) of three qPCR reactions performed on each sample.

**Table 4:** Probabilities of infection by 0, 1, 2 or 3 occlusion derived virions in each well of end-point dilution assays calculated following the Poisson probability distribution.

		T12			T24	
	Repetition 1	Repetition 2	Repetition 3	Repetition 1	Repetition 2	Repetition 3
No. positive wells/Total ^1^	11/88	8/88	8/88	7/88	11/88	13/88
Probability (0)	0.875	0.909	0.909	0.920	0.875	0.852
Probability (1)	0.117	0.0866	0.0866	0.0763	0.117	0.136
Probability (2)	0.00780	0.00413	0.00413	0.00316	0.00780	0.0109
Probability (3)	0.000347	0.000131	0.000131	0.0000874	0.000347	0.000580

^1^ Positive wells contained at least one cell with pathological signs of NPV infection (OBs in the nucleus). Probabilities were calculated following the Poisson probability distribution of the cells in a particular well being infected by one, two or three ODVs, respectively, i.e., probability *p* = (e^−µ^ * µ*^x^*)/*x*!, where µ is the rate parameter estimated from the observed proportion of non-infected wells (P_0_ = e^−µ^) and *x* is the number of infecting ODVs (1, 2, 3...) [15].

**Table 5:** LC_50_ values and relative potencies of HearMNPV, HearSNPV, mixed-virus preparations T12 and T24 and the OB mixture of 50% HearSNPV OBs + 50% HearMNPV OBs in second instars of *H. armigera, S. frugiperda* and *M. brassicae*. The expected LC_50*m*_ values were calculated using Tabashnik’s equation [49].

		LC_50_	Relative	95% Fiducial Limits		Expected
Host	Virus Treatment ^1^	(OBs/mL)	Potency	Low	High	LC_50*m*_
*H. armigera*	HearMNPV	1.0 × 10^5^	1	-	-	-
	HearSNPV	1.5 × 10^4^	6.6	2.5	17.2	-
	T12 mixed-virus	2.2 × 10^4^	4.5	1.9	10.8	3.1 × 10^4^
	T24 mixed-virus	3.5 × 10^4^	2.9	1.5	5.9	2.4 × 10^4^
	50% SNPV + 50% MNPV	3.0 × 10^4^	3.4	1.6	7.1	2.7 × 10^4^
*S. frugiperda*	HearMNPV	2.0 × 10^7^	1	-	-	-
	HearSNPV	-	-	-	-	-
	T12 mixed-virus	3.8 × 10^7^	0.5	0.3	0.9	3.4 × 10^7^
	T24 mixed-virus	3.6 × 10^7^	0.6	0.3	0.9	4.7 × 10^7^
	50% SNPV + 50% MNPV	4.2 × 10^7^	0.5	0.3	0.8	4.0 × 10^7^
*M. brassicae*	HearMNPV	2.0 × 10^5^	1	-	-	-
	HearSNPV	-	-	-	-	-
	T12 mixed-virus	3.9 × 10^5^	0.5	0.3	0.9	3.3 × 10^5^
	T24 mixed-virus	4.1 × 10^5^	0.5	0.2	0.9	4.6 × 10^5^
	50% SNPV + 50% MNPV	5.5 × 10^5^	0.4	0.2	0.7	3.9 × 10^5^

^1^ Relative potencies were calculated as the ratio of LC_50_ values relative to the HearMNPV. It was not possible to lethally infect *S. frugiperda* or *M. brassicae* larvae with HearSNPV even at the highest inoculum concentrations tested, namely 1.5 × 10^9^ and 3.1 × 10^6^ OBs/mL, respectively.

**Table 6:** Relative prevalence (%) of HearSNPV genomes quantified by qPCR in progeny OBs collected following an initial infection cycle (P1) of T0, T48 and T72 mixed-virus preparations and five steps of serial passage (P1–P5) of the T12 and T24 preparations and the OB mixture comprising 50% HearSNPV OBs + 50% HearMNPV OBs in *H. armigera*, *S. frugiperda* and *M. brassicae* larvae.

Host Species	Virus Sample	P0 ^1^	P1	P2	P3	P4	P5
*H. armigera*	T0	96.76	98.92147	-	-	-	-
	T12	41.01	99.91097	100.00	100.00	100.00	100.00
	T24	57.34	96.46719	100.00	100.00	100.00	100.00
	T48	3.20	63.90424	-	-	-	-
	T72	4.41	88.43874	-	-	-	-
	50% SNPV + 50% MNPV	50.00	97.88138	100.00	100.00	100.00	100.00
*S. frugiperda*	T0	96.76	0.00008	-	-	-	-
	T12	41.01	0.00129	0.00003	0.00004	0.00003	0.00006
	T24	57.34	0.00106	0.00001	0.00002	0.00001	0.00001
	T48	3.20	0.00002	-	-	-	-
	T72	4.41	0.00017	-	-	-	-
	50% SNPV + 50% MNPV	50.00	0.00013	0.00002	0.00001	0.00002	0.00002
*M. brassicae*	T0	96.76	0.00084	-	-	-	-
	T12	41.01	0.01113	0.01987	0.00166	0.00348	0.00569
	T24	57.34	0.00109	0.00311	0.00071	0.00026	0.00018
	T48	3.20	0.02164	-	-	-	-
	T72	4.41	0.02384	-	-	-	-
	50% SNPV + 50% MNPV	50.00	0.02933	0.00348	0.00087	0.00108	0.00064

^1^ The P0 inocula had an identical initial composition in all hosts tested.

## Data Availability

All the data in the present article are presented in Figure 1, Figure 2, Figure 3, Figure 4 and Figure 5 and Table 1, Table 2, Table 3, Table 4, Table 5 and Table 6 of the article.

## Supplementary Materials

The following are available online at https://www.mdpi.com/article/10.3390/v14040687/s1. Figure S1: Logarithm of copy number of HearSNPV and HearMNPV genomes determined by qPCR of progeny occlusion bodies.

## References

[1] Arnemann J.A., Roxburgh S., Walsh T., Guedes J., Gordon K., Smagghe G., Tay W.T. (2019). Multiple incursion pathways for *Helicoverpa armigera* in Brazil show its genetic diversity spreading in a connected world. Sci. Rep..

[2] Mironidis G.K., Kapantaidaki D., Bentila M., Morou E., Savopoulou-Soultani M., Vontas J. (2013). Resurgence of the cotton bollworm *Helicoverpa armigera* in northern Greece associated with insecticide resistance. Insect Sci..

[3] Sun X. (2015). History and current status of development and use of viral insecticides in China. Viruses.

[4] Grzywacz D., Moore S., Lacey L.A. (2017). Production, formulation, and bioassay of baculoviruses for pest control. Microbial Control of Insect and Mite Pests.

[5] Harrison R.L., Herniou E.A., Jehle J.A., Theilmann D.A., Burand J.P., Krell P.J., van Oers M.M., Mowery J.D. (2019). ICTV virus taxonomy profile: Baculoviridae. J. Gen. Virol..

[6] Sajjan D.B., Hinchigeri S.B. (2016). Structural organization of baculovirus occlusion bodies and protective role of multilayered polyhedron envelope protein. Food Environ. Virol..

[7] Rohrmann G.F. (2019). Baculovirus Molecular Biology.

[8] Erlandson M.A., Toprak U., Hegedus D.D. (2019). Role of the peritrophic matrix in insect-pathogen interactions. J. Ins. Physiol..

[9] Passarelli A.L. (2011). Barriers to success: How baculoviruses establish efficient systemic infections. Virology.

[10] Williams T., Hajek A.E., Shapiro-Ilan D.I. (2018). Viruses. Ecology of Invertebrate Diseases.

[11] Erlandson M. (2009). Genetic variation in field populations of baculoviruses: Mechanisms for generating variation and its potential role in baculovirus epizootiology. Virol. Sin..

[12] Masson T., Fabre M.L., Pidre M.L., Niz J.M., Berretta M.F., Romanowski V., Ferrelli M.L. (2021). Genomic diversity in a population of Spodoptera frugiperda nucleopolyhedrovirus. Infect. Genet. Evol..

[13] Clavijo G., Williams T., Muñoz D., Caballero P., López-Ferber M. (2010). Mixed genotype transmission bodies and virions contribute to the maintenance of diversity in an insect virus. Proc. R. Soc. B Biol. Sci..

[14] Bernal A., Simón O., Williams T., Muñoz D., Caballero P. (2013). A Chrysodeixis chalcites single nucleopolyhedrovirus population from the Canary Islands is genotypically structured to maximize survival. Appl. Environ. Microbiol..

[15] Beperet I., Simón O., López-Ferber M., van Lent J., Williams T., Caballero P. (2021). Mixtures of insect pathogenic viruses in a single virion: Towards the development of custom designed insecticides. Appl. Environ. Microbiol..

[16] Bull J.C., Godfray H.C.J., O’Reilly D.R. (2001). Persistence of an occlusion-negative recombinant nucleopolyhedrovirus in Trichoplusia ni indicates high multiplicity of cellular infection. Appl. Environ. Microbiol..

[17] Cory J.S., Green B.M., Paul R.K., Hunter-Fujita F. (2005). Genotypic and phenotypic diversity of a baculovirus population within an individual insect host. J. Invertebr. Pathol..

[18] Baillie V.L., Bouwer G. (2013). The effect of inoculum dose on the genetic diversity detected within *Helicoverpa armigera* nucleopolyhedrovirus populations. J. Gen. Virol..

[19] Chateigner A., Bézier A., Labrousse C., Jiolle D., Barbe V., Herniou E.A. (2015). Ultra deep sequencing of a baculovirus population reveals widespread genomic variations. Viruses.

[20] Xu X., Chen Y., Zhao Y., Liu X., Dong B., Jones I.M., Chen H. (2013). Baculovirus superinfection: A probable restriction factor on the surface display of proteins for library screening. PLoS ONE.

[21] Beperet I., Irons S., Simón O., King L.A., Williams T., Possee R.D., López-Ferber M., Caballero P. (2014). Superinfection exclusion in alphabaculovirus infections is concomitant with actin reorganization. J. Virol..

[22] Gettig R.R., McCarthy W.J. (1982). Genotypic variation among isolates of Heliothis spp. nuclear polyhedrosis viruses from different geographical regions. Virology.

[23] Figueiredo E., Muñoz D., Escribano A., Mexia A., Vlak J.M., Caballero P. (1999). Biochemical identification and comparative insecticidal activity of nucleopolyhedrovirus pathogenic for Heliothis armigera (Lep. Noctuidae) larvae. J. Appl. Entomol..

[24] Ogembo J.G., Kunjeku E.C., Sithanantham S. (2005). A preliminary study on the pathogenicity of two isolates of nucleopolyhedroviruses infecting the African bollworm, *Helicoverpa armigera* (Lepidoptera: Noctuidae). Int. J. Trop. Ins. Sci..

[25] Zhang C.X., Ma X.C., Guo Z.J. (2005). Comparison of complete genome sequence between C1 and G4 isolates of the *Helicoverpa armigera* single nucleocapsid nucleopolyhedrovirus. Virology.

[26] Williams C.F., Payne C.C. (1984). The susceptibility of Heliothis armigera to three nuclear polyhedrosis viruses. Ann. Appl. Biol..

[27] Sun X.L., Zhang G.Y. (1994). Comparison of four Heliothis armigera NPV isolates. Virol. Sin..

[28] Figueiredo E., Muñoz D., Murillo R., Mexia A., Caballero P. (2009). Diversity of Iberian nucleopolyhedrovirus wild-type isolates infecting *Helicoverpa armigera* (Lepidoptera: Noctuidae). Biol. Control.

[29] Ogembo J.G., Caoili B.L., Shikata M., Chaeychomsri S., Kobayashi M., Ikeda M. (2009). Comparative genomic sequence analysis of novel *Helicoverpa armigera* nucleopolyhedrovirus (NPV) isolated from Kenya and three other previously sequenced Helicoverpa spp. NPV. Virus Genes.

[30] Rowley D.L., Popham H.J., Harrison R.L. (2011). Genetic variation and virulence of nucleopolyhedroviruses isolated worldwide from the heliothine pests *Helicoverpa armigera*, Helicoverpa zea, and Heliothis virescens. J. Invertebr. Pathol..

[31] Arrizubieta M., Simón O., Williams T., Caballero P. (2015). Genomic sequence of five *Helicoverpa armigera* nucleopolyhedrovirus genotypes from Spain that differ in their insecticidal properties. Genome Anounc..

[32] Raghavendra A.T., Jalali S.K., Ojha R., Shivalingaswamy T.M., Bhatnagar R. (2017). Whole genome sequence and comparative genomic sequence analysis of *Helicoverpa armigera* nucleopolyhedrovirus (HearNPV-L1) isolated from India. VirusDisease.

[33] Singh R., Jagadish K.S., Tak K.R., Peter A. (2019). Genetic diversity among different geographical isolates of the gram pod borer, *Helicoverpa armigera* (Hübner)(Lepidoptera: Noctuidae) nucleopolyhedrosis virus (Hear NPV). Egypt. J. Biol. Pest Contr..

[34] Eroglu G.B., Inan C., Nalcacioglu R., Demirbag Z. (2020). Genome sequence analysis of a *Helicoverpa armigera* single nucleopolyhedrovirus (HearNPV-TR) isolated from Heliothis peltigera in Turkey. PLoS ONE.

[35] Gröner A., Federici B.B., Granados R.R. (1986). Specificity and safety of baculoviruses, In The Biology of Baculoviruses: Biological Properties and Molecular Biology.

[36] Tang P., Zhang H., Li Y., Han B., Wang G., Qin Q., Zhang Z. (2012). Genomic sequencing and analyses of HearMNPV—A new multinucleocapsid nucleopolyhedrovirus isolated from *Helicoverpa armigera*. Virol. J..

[37] Rovesti L., Crook N.E., Winstanley D. (2000). Biological and biochemical relationships between the nucleopolyhedroviruses of Mamestra brassicae and Heliothis armigera. J. Invertebr. Pathol..

[38] Tompkins G.J., Dougherty E.M., Adams J.R., Diggs D. (1988). Changes in the virulence of nuclear polyhedrosis viruses when propagated in alternate noctuid (Lepidoptera: Noctuidae) cell lines and hosts. J. Econ. Entomol..

[39] Belda I.M., Beperet I., Williams T., Caballero P. (2019). Genetic variation and biological stability of two closely related alphabaculoviruses during serial passage in permissive and semi-permissive heterologous hosts. Viruses.

[40] Moore S.D., Bouwer G., Pittway T.M. (2004). Evaluation of *Helicoverpa armigera* nucleopolyhedrosis virus (HearNPV) for control of *Helicoverpa armigera* (Lepidoptera: Noctuidae) on citrus in South Africa. Biocontrol Sci. Technol..

[41] Arrizubieta M., Williams T., Caballero P., Simón O. (2014). Selection of a nucleopolyhedrovirus isolate from *Helicoverpa armigera* as the basis for a biological insecticide. Pest Manag. Sci..

[42] Greene G.L., Leppla N.C., Dickerson W.A. (1976). Velvetbean caterpillar: A rearing procedure and artificial medium. J. Econ. Entomol..

[43] Mclntosh A.H., Ignoffo C.M. (1981). Replication and infectivity of the single-embedded nuclear polyhedrosis virus, Baculovirus heliothis, in homologous cell lines. J. Invertebr. Pathol..

[44] Hughes P.R., Wood H.A. (1981). A synchronous peroral technique for the bioassay of insect viruses. J. Invertebr. Pathol..

[45] Le Ora Software (1987). POLO-PC a User’s Guide to Probit or Logit Analysis.

[46] Crawley M.J. (1993). GLIM for Ecologists.

[47] Guo Z.J., An S.H., Wang D., Liu Y.H., Kumar V.S., Zhang C.X. (2005). Characterization of Ha29, a specific gene for *Helicoverpa armigera* single-nucleocapsid nucleopolyhedrovirus. J. Biochem. Mol. Biol..

[48] Jamovi Statistical Software 2021. Jamovi Project v.2.3.0.0. https://www.jamovi.org.

[49] Tabashnik B.E. (1992). Evaluation of synergism among Bacillus thuringiensis toxins. Appl. Env. Microbiol..

[50] Beperet-Arive I. (2014). Regulation of Multiple Infection in Alphabaculoviruses: Critical Factors that Determine Success. Doctoral Thesis.

[51] Williams T., López-Ferber M., Caballero P. (2022). Nucleopolyhedrovirus coocclusion technology: A new concept in the development of biological insecticides. Front. Microbiol..

[52] Makalliwa G.A., Wang X., Zhang H., Zhang N., Chen C., Li J., Deng F., Wang H., Wang M., Hu Z. (2018). HearNPV pseudotyped with PIF1, 2, and 3 from MabrNPV: Infectivity and complex stability. Virol. Sin..

[53] Torres-Vila L.M., Rodríguez-Molina M.C., Lacasa-Plasencia A. (2003). Impact of *Helicoverpa armigera* larval density and crop phenology on yield and quality losses in processing tomato: Developing fruit count-based damage thresholds for IPM decision-making. Crop Protec..

[54] Rojas J.C., Wyatt T.D., Birch M.C. (2000). Flight and oviposition behavior toward different host plant species by the cabbage moth, Mamestra brassicae (L.) (Lepidoptera: Noctuidae). J. Insect Behav..

[55] Cartea M.E., Francisco M., Lema M., Soengas P., Velasco P. (2010). Resistance of cabbage (Brassica oleracea capitata group) crops to Mamestra brassicae. J. Econ. Entomol..

[56] CABI (2021). Invasive Species Compendium. Datasheet *Helicoverpa armigera* (Cotton Bollworm). https://www.cabi.org/isc/datasheet/26757#tohostsOrSpeciesAffected.

[57] Acharya R., Akintola A.A., Malekera M.J., Kamulegeya P., Nyakunga K.B., Mutimbu M.K., Shrestha Y.K., Hemayet J.S., Hoat T.X., Dao H.T. (2021). Genetic relationship of fall armyworm (Spodoptera frugiperda) populations that invaded Africa and Asia. Insects.

[58] Sanches M.M., Guimarães G.C., Sihler W., Souza M.L. (2021). Successful co-infection of two different baculovirus species in the same cell line reveals a potential strategy for large in vitro production. Braz. J. Microbiol..

[59] Sanches M.M., Sihler W., Silva C.E.P., Guimarães G.C., Benito N.P., Sosa-Gómez D.R., de Souza M.L. (2019). Characterization of a Chrysodeixis includens nucleopolyhedrovirus isolate from Brazilian cerrado and assessment of its co-infection with Anticarsia gemmatalis multiple nucleopolyhedrovirus. Braz. Arch. Biol. Technol..

[60] (2019). AgBiTech launches lepidopteran biocontrol options. Outlooks Pest Manag..

[61] Pavan O.H.O., Ribeiro H.C.T. (1989). Selection of a baculovirus strain with a bivalent insecticidal activity. Mem. Inst. Oswaldo Cruz.

[62] Kolodny-Hirsch D.M., Van Beek N.A.M. (1997). Selection of a morphological variant of Autographa californica nuclear polyhedrosis virus with increased virulence following serial passage in Plutella xylostella. J. Invertebr. Pathol..

[63] Graillot B., Blachère-López C., Besse S., Siegwart M., López-Ferber M. (2016). Host range extension of Cydia pomonella granulovirus: Adaptation to oriental fruit moth, Grapholita molesta. BioControl.

[64] Shapiro M., Martignoni M.E., Cunningham J.C., Goodwin R.H. (1982). Potential use of the saltmarsh caterpillar as a production host for nucleopolyhedrosis viruses. J. Econ. Entomol..

[65] Kitchin D., Bouwer G. (2018). Significant differences in the intra-host genetic diversity of *Helicoverpa armigera* nucleopolyhedrovirus dnapol after serial in vivo passages in the same insect population. Arch. Virol..

[66] Arrizubieta M., Simón O., Williams T., Caballero P. (2015). A novel binary mixture of *Helicoverpa armigera* single nucleopolyhedrovirus genotypic variants has improved insecticidal characteristics for control of cotton bollworms. Appl. Environ. Microbiol..

[67] Simón O., Williams T., Caballero P., López-Ferber M. (2006). Dynamics of deletion genotypes in an experimental insect virus population. Proc. R. Soc. B Biol. Sci..

[68] Zwart M.P., Pijlman G.P., Sardanyés J., Duarte J., Januário C., Elena S.F. (2013). Complex dynamics of defective interfering baculoviruses during serial passage in insect cells. J. Biol. Phys..

[69] Hitchman R.B., Hodgson D.J., King L.A., Hails R.S., Cory J.S., Possee R.D. (2007). Host mediated selection of pathogen genotypes as a mechanism for the maintenance of baculovirus diversity in the field. J. Invertebr. Pathol..

[70] Hou D., Chen X., Zhang L.K. (2016). Proteomic analysis of Mamestra brassicae nucleopolyhedrovirus progeny virions from two different hosts. PLoS ONE.

[71] Aguirre E., Beperet I., Williams T., Caballero P. (2021). Generation of variability in Chrysodeixis includens nucleopolyhedrovirus (ChinNPV): The role of a single variant. Viruses.

[72] Clavijo G., Williams T., Muñoz D., López-Ferber M., Caballero P. (2009). Entry into midgut epithelial cells is a key step in the selection of genotypes in a nucleopolyhedrovirus. Virol. Sin..

[73] Zwart M.P., Hemerik L., Cory J.S., de Visser J.A.G.M., Bianchi F.J.J.A., Van Oers M.M., Vlak J.M., Hoekstra R.F., Van der Werf W. (2009). An experimental test of the independent action hypothesis in virus–insect pathosystems. Proc. R. Soc. B Biol. Sci..

[74] Mu J., van Lent J.W., Smagghe G., Wang Y., Chen X., Vlak J.M., van Oers M.M. (2014). Live imaging of baculovirus infection of midgut epithelium cells: A functional assay of per os infectivity factors. J. Gen. Virol..

[75] Wang X., Shang Y., Chen C., Liu S., Chang M., Zhang N., Hu H., Zhang F., Zhang T., Wang Z. (2019). Baculovirus per os infectivity factor complex: Components and assembly. J. Virol..

[76] López-Ferber M., Simón O., Williams T., Caballero P. (2003). Defective or effective? Mutualistic interactions between virus genotypes. Proc. R. Soc. B Biol. Sci..

[77] Zwart M.P., Elena S.F. (2015). Matters of size: Genetic bottlenecks in virus infection and their potential impact on evolution. Annu. Rev. Virol..

[78] Washburn J.O., Trudeau D., Wong J.F., Volkman L.E. (2003). Early pathogenesis of Autographa californica multiple nucleopolyhedrovirus and Helicoverpa zea single nucleopolyhedrovirus in Heliothis virescens: A comparison of the ‘M’ and ‘S’ strategies for establishing fatal infection. J. Gen. Virol..

