# Supplementary material for: Coocclusion of Helicoverpa armigera Single Nucleopolyhedrovirus (HearSNPV) and Helicoverpa armigera Multiple Nucleopolyhedrovirus (HearMNPV): Pathogenicity and Stability in Homologous and Heterologous Hosts
Source: Viruses. 2022 Mar 26;14(4):687. doi: 10.3390/v14040687 (PMC9025457; doi:10.3390/v14040687)
Supplement: Supplementary file 1 [file viruses-14-00687-s001.zip › viruses-1627862-supplementary.pdf]

Supplemental figure: S1. Logarithm of copy number of HearSNPV and HearMNPV genomes determined by qPCR of progeny occlusion bodies.

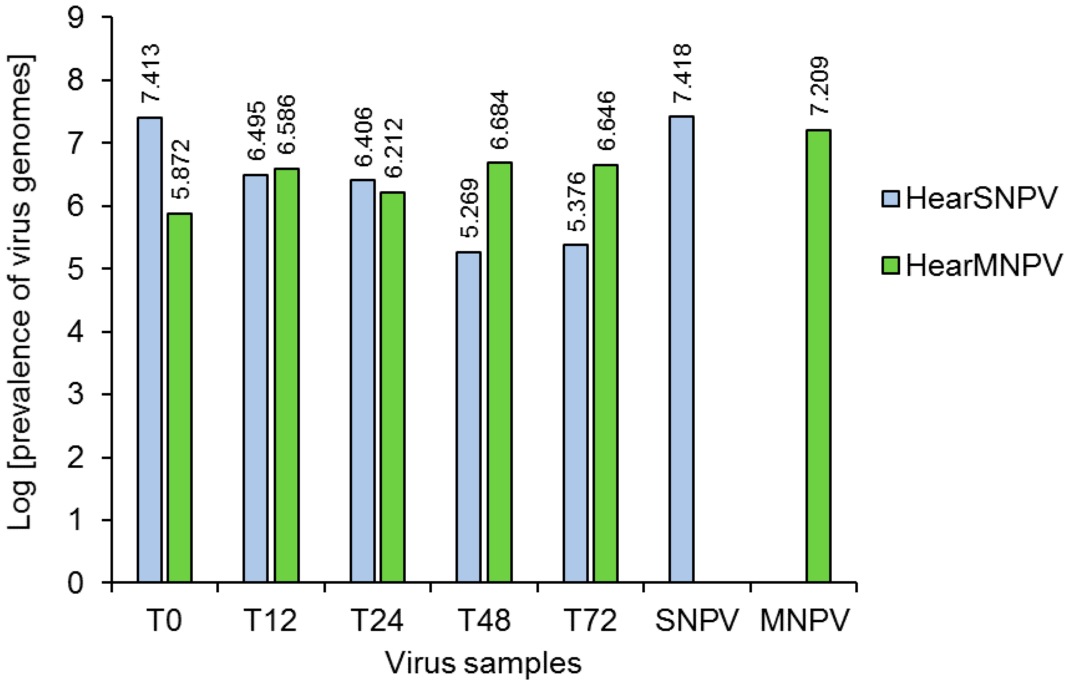

Figure S1. Logarithm of copy number of HearSNPV (blue columns) and HearMNPV (green columns) genomes determined by qPCR of progeny occlusion bodies obtained after initial infection of *Helicoverpa armigera* larvae with an LC_{90} concentration of HearMNPV followed at intervals of 0 h (T0), 12 h (T12), 24 h (T24), 48 h (T48) and 72 h (T72) by superinfection with an LC_{90} concentration of HearSNPV occlusion bodies. Values above columns indicate logarithms of the prevalence of virus genomes (copy number).
